# Evaluation of Aqueous Leaf Extract of *Cardiospermum halicacabum* (L.) on Fertility of Male Rats

**DOI:** 10.1155/2015/175726

**Published:** 2015-05-07

**Authors:** L. Dinithi. C. Peiris, M. A. T. Dhanushka, T. A. H. D. G. Jayathilake

**Affiliations:** ^1^Department of Zoology, University of Sri Jayewardenepura, 10250 Nugegoda, Sri Lanka; ^2^Environemtnal Management Division, Abans Environmental Pvt. Ltd., Colombo 5, Sri Lanka

## Abstract

Treatment with 100 mg/kg and 200 mg/kg body weight of aqueous leaf extract (ALE) of *Cardiospermum halicacabum* for 30 days produced a significant dose dependent increase in the sperm counts and sperm motility in both caput and cauda regions. Further, significant increase in serum testosterone level was evident at all applied doses. However, no significant changes in the weight of sex organs were observed. Aqueous leaf extract also increased the number of females impregnated, number of implantations, and number of viable fetuses while decreasing the total number of resorption sites in the pregnant females. However, the total cholesterol level in the serum remained unchanged and there were no records on renotoxicity; nevertheless ALE exhibited a hepatoprotective effect. It was concluded that aqueous leaf extract of *Cardiospermum halicacabum* enhanced sperm concentration, motility, and testosterone, leading to positive results in fertility.

## 1. Introduction 

In Sri Lanka, thousands of plants species are known to have medicinal values. Different parts of these plant species are still widely used in traditional Ayurveda and Siddha-Ayrveda medicine practices to cure various diseases. More than 60–70% of the rural population still relies on traditional medicine for their primary health care needs [1]. Recently scientific interest in medicinal plants has burgeoned due to increased efficiency of plant derived drugs and raising concern about the side effects of modern medicine.* Cardiospermum halicacabum *L. belonging to family Sapindaceae is one such plant widely used by traditional practitioners in Sri Lanka to cure various ailments.

*Cardiospermum halicacabum *(Welpenela in Sinhala; balloon vine in English) is an annual or sometimes perennial herb, which is very common in the low country of Sri Lanka. It is a small delicate, smooth, climber and the whole plant has been used for several centuries in the treatment of rheumatism, stiffness of limbs, and snakebites [2]. A decoction of root is given for bleeding piles. The roots are used for nervous diseases [3] and as a diaphoretic, diuretic, emetic, emmenagogue, laxative, refrigerant, stomachic, and sudorific [4]. Fresh leaf juice is given for asthma patients and it can also reduce obesity. The leaves also are used as one of the ingredients in a medicine for abnormal suppression of menstrual cycle. Leaves boiled in oil such as castor oil are applied over rheumatic pain swellings and tumors of various kinds [5]. Several studies suggest that ethanol extract of leaves possess antidiabetes [6, 7] and antibacterial [8, 9] activities. Phytochemical constituents such as flavones, aglycones, triterpenoids, glycosides, and a range of fatty acids and volatile ester have been reported from the various extracts of this plant [9].

Further it has been recorded in traditional Sri Lankan medicinal system that* C. halicacabum* leaves are used to treat male infertility. However, this has not been scientifically proven or documented. Male infertility is an increasing concern worldwide including Sri Lanka. Recent findings suggest that about 48.5 million couples worldwide are unable to have a child after 5 years [10]. Hence, the present study was carried out to determine the fertility effects and possible side effects of aqueous leaf extract of* C. halicacabum*.

## 2. Materials and Methods

### 2.1. Plant Material

Fresh* Cardiospermum halicacabum *leaves were collected in the morning (9.00 a.m.-10.00 a.m.) from mature, healthy plants grown in home gardens of Colombo, Sri Lanka, between February and April in 2014 and were identified and authenticated by Professor B.M.P Singhakumara, Department of Forestry and Environment Science, University of Sri Jayewardenepura. A voucher specimen (SP number DP/02) was deposited in the Department of Zoology, University of Sri Jayewardenepura.

### 2.2. Preparation of Leaves Extract

Leaves (60 g) of* Cardiospermum halicacabum* L. were dried in the shade. Subsequently, the dried leaves were powdered to get a course powder. About 25 g of dry powder was mixed with 200 mL distilled water and refluxed using continuous percolation method using Soxhlet apparatus. The extractions were continued for 72 h at 50°C. The extract were filtered and concentrated to a dry mass by a vacuum distillation method. The extract was stored in desiccators and was dissolved in distilled water to prepare required dosages in 1 mL solution.

### 2.3. Animals

Wistar male rats weighing between 200 g and 250 g were used for this study. The animals were purchased from the Medical Research Institute, Colombo, Sri Lanka, and housed in the animal house of Department of Biochemistry, University of Sri Jayewardenepura. Six rats were taken for each group. The rats were acclimated to the laboratory environment for 7 days prior to the study. Animal house was well maintained under standard hygienic condition at (26 ± 2)°C, relative humidity of 44%–55%, 12 h day and night cycle, and with food and water ad libitum. The animal experiment was performed as per committee for the purpose of control and supervision of experiments on animal norms after obtaining institutional animal ethics committee clearance.

### 2.4. Experimental Design

Thirty healthy male rats (14–20 weeks old) were divided randomly into to 3 equal groups of 10 animals each and orally administered with different test materials using feeding tube for consecutive 30 days (between 9.00 am and 10.00 am). Animals were gavaged with aqueous leaf extract (ALE) in the following manner:

 Group A: 1 mL of 200 mg/kg ALE (*n* = 10)—higher dose;
 Group B: 1 mL of 100 mg/kg ALE (*n* = 10)—lower dose;
 Group C: 1 mL of distilled water (*n* = 10)—negative control.

Animals from each group (both treated and control groups) were sacrificed on day 31 using an overdose of ether anesthesia.

### 2.5. Effects of ALE on Epididymal and Testicular Weight

Upon scarification, left and right epididymes and testes were removed and cleaned to eliminate all the fat bodies. Epididymal and testicular weight were recorded using an electric balance (Mettler, AE 100, UK).

### 2.6. Preparation of Sperm Suspension

Epididymis was separated carefully from testis and divided into 2 segments: caput (the head) and cauda (the tail). These parts from both epididymes were put into different vials and minced with scissors and gently homogenized with 2 mL mammalian saline solution. Approximately 100 *µ*L of this concentrated sperm suspension was pipetted out into another vial and mixed with 900 *µ*L of mammalian saline solution. Evaluation of sperm count, an hour after the sperm diffusion in the solution a 10 *μ*L aliquot of the epididymal sperm suspension was transferred to each counting chamber of the hemocytometer and allowed to stand for 5 min. Total sperm count of corpus and cauda epididymis regions were estimated using a light microscope at ×40 magnification. Sperm counts were expressed as million/mL of suspension.

### 2.7. Effects ALE on Sperm Motility Parameters and Total Epididymal Sperm Count

A sample drop from different epididymal areas was taken and percentage motile spermatozoa were estimated (approximately 200 cells) for each concentration and at each time points by a single observation under phase contrast optics (×200; Olympus Corporation, Japan). Spermatozoa which represent various motility patterns including immotile sperm, slightly motile sperm, twitching sperm, slow moving sperm, highly motile sperm were scored. Total sperm number was determined in 5 replicates.

### 2.8. Effects of ALE on Serum Testosterone Levels

Three groups of rats were taken (*n* = 10 per group) and treated orally in the following manner: group A with 10 mL of 100 mg/kg ALE; group B with 1 mL of 200 mg/kg ALE; and group C with 1 mL of DW per day for 30 consecutive days. On day 31, blood samples were collected from the tails and serum was separated. Subsequently serum testosterone level was measured using Enzyme Immunoassay Method (EIA). The EIA kit was obtained from Immunometrics (London, UK).

### 2.9. Effects of ALE on Male Fertility

Separate 3 groups of animals (*n* = 10 per group) were gavaged with 2 different doses of AFE and the control for 30 consecutive days. On day 31 of treatment, males were paired overnight with a prooestrous female (at 16:30–17:00 pm). The following morning (08:00 a.m.–08:30 a.m.), successful mating was confirmed by the presence of sperm in the vaginal smear. If spermatozoa were present, their numbers were estimated (in duplicate) using an improved Neubauer haemocytometer and gross morphology was noted by microscopic examination (×200).

On day 14 of gestation, the female rats were laporatomized under mild ether anesthesia under aseptic conditions. Upon laparotomy, number of dead and live uterine implants and number of corpora lutea in both uterine horns were determined. Further, the width and the length of implants were recorded. At the end of the gestation period, number of live and dead pups was recorded.

### 2.10. Effects of ALE on Lipid Profile

Three groups of rats were taken (*n* = 10 per group) and treated orally in the following manner: group A with 1 mL of 100 mg/kg ALE; group B with 1 mL of 200 mg/kg ALE; and group C with 1 mL of DW per day for 30 consecutive days. On day 31, blood samples were collected from the tails and serum was separated. TC (total cholesterol), HDL-cholesterol (high-density cholesterol), LDL-cholesterol, and triglycerides concentrations were measured using levels using respective Randox enzyme kits (Randox Laborotories, Antrim, UK) and a spectrophotometer.

### 2.11. Toxicological Studies

Thirty (*n* = 10/group) rats were orally treated (between 9.00 a.m. and 10.00 a.m.) with 1 mL of 500 mg/kg of ALE, 200 mg/kg ALE or 1 mL of DW for 30 consecutive days. Animals were observed daily between 11.00 a.m. and 12 noon for any overt sign of toxicity (diarrhoea, salivation, lachrymation, tremors, ataxia, loss of fur, change of fur colour, postural abnormalities, or behavioural changes), stress (fur erection and exophthalmia), and aversive behaviours, during the treatment period. Percentage of body weight gain and food and water intake was determined during the study period.

On day 31 after treatment, approximately 2 mL blood was collected from heart puncture using aseptic precautions and divided into two equal parts. Blood was collected and was allowed to clot for 25–30 min at room temperature (28–30°C) and centrifuged at 3000 rpm for 5 min to separate serum. Serum samples were analyzed for concentrations of urea, creatinine, and alanine aminotransferase (ALT), and aspartate aminotransferase (AST) concentrations were determined using Biolabo kits (Biolabo Reagent, Maizy, France). Absorption was measured using a spectrophotometer (Labomed, Inc., Los Angeles, USA), respectively, at 500 nm and 340 nm using respective Randox enzyme kits (Randox Laborotories, Antrim, UK) and a spectrophotometer. Gamma-glutamyltransferase (GGT) concentrations were determined using GGT-enzymatic assay kit (Xperss Bio Products, USA).

After drawing blood, mice were sacrificed with and overdose of ether and weighed. Parts of kidney and liver were fixed in Bouin's solution and histological sections were prepared and stained with hemotoxylin and eosin stains [11]. Subsequently, the slides were examined under high power (×200) using a phase contrast microscope (Nikon Corporation, Tokyo, Japan) for pathological changes.

### 2.12. Statistical Analysis

All data were expressed as mean SEM statistical analysis was performed by using one way analysis of variance (one way ANOVA) and differences between pairs of means were made by using the student's Newman-Keules test.

## 3. Results

### 3.1. Epididymal, Testicular, and Seminal Vesicle Weights

Treatment with 2 doses of ALE did not significantly (*p* > 0.05) change the organ weights of epididymes, testes, and seminal vesicles compared to the control (Table 1).

### 3.2. Total Sperm Count

Total sperm count in cauda epididymis showed significant increase in treatment group A (312.53 ± 12.37) and group B (294.94 ± 5.03) when compared to the control (224.54 ± 7.92). Similarly, in corpus region, the total sperm count of treatment group A (245.25 ± 2.74) and group B (228.47 ± 8.90) increased significantly compared to the control (104.97 ± 7.54). See Table 2.

### 3.3. Sperm Motility

Percentage immotile sperm count in both treated groups reduced significantly (*p* < 0.05) when compared to the control group. Immotile sperm count in caput epididymis of group A (40.25 ± 2.74) was decreased by 13% and by 6% in group B (44.47 ± 5.38) compared to the control (46.93 ± 1.29). Further, there was a significant (*p* < 0.05) decrease of twitching sperm number in caput region in animals treated with 100 mg/kg ALE (14.62 ± 3.26) and in animals treated with 200 mg/kg ALE (15.89 ± 2.05) compared to the control (24.57 ± 4.43). Similarly, percentage of motile sperm increased significantly (*p* < 0.01) in groups A (28.99 ± 3.26) and B (38.60 ± 5.03) compared to the control (18.44 ± 1.26).

In cauda region, percentages of immotile sperm number and twitching sperm number did not show a significant difference when compared to the control (Table 3). However, the percentage motile sperm number in cauda region of group A (188.15 ± 3.39) and group B (157.58 ± 4.41) showed a significant (*p* < 0.01) increase compared to the control (106.79 ± 2.26).

### 3.4. Serum Testosterone Level

The aqueous extract of* C. halicacabum *leaves (200 mg/kg and 100 mg/kg) repeated treatment for 30 days caused significant (*p* < 0.05) increase in testosterone level in male rats (Table 4).

### 3.5. Fertility Test

The results presented in Table 5 shows that oral administration of aqueous extract of* C. halicacabum* at dose (200 mg/kg body weight) for 30 days to male rats had significant (*p* < 0.01) increase on the number of females impregnated by them. The number of implantations and number of viable fetuses were significantly (*p* < 0.01) increased in female rats impregnated by males ingested ALE. Also the number of resorptions sites was significantly (*p* < 0.05) decreased in females impregnated by males ingested (200 mg/kg body weight).

### 3.6. Effects of ALE on Lipid Profile

The treatment with the high and low doses of ALE did not significantly (*p* > 0.05) change the concentration of total cholesterol (group A: 90 ± 4.87; group B: 92 ± 3.65; group C: 91 ± 2.31), HDL-cholesterol (group A: 20.60 ± 3.38; group B: 20.20 ± 2.20, group C: 20.53 ± 2.64), LDL-cholesterol (group A: 44 ± 4.12; group B: 44 ± 3.25; group C: 44 ± 3.62), and triglycerides (group A: 112 ± 3.45; group B: 111 ± 51; group C: 112 ± 5.31) in the serum as compared with the control (data not shown).

### 3.7. Toxicological Studies

Oral treatment of ALE did not induce any overt signs of clinical toxicity or stress in acute or chronic terms. Further, treatment with ALE showed no Reno toxicity (serum urea, creatinine, and total protein) and hepatotoxicity (ALT, AST, and GGT).

Test for the qualitative determination for the alkaline phosphates and acid phosphates enzymes in serum showed significant (*p* < 0.05) decrease in treatment groups A and B when compared to control (Table 6).

## 4. Discussion

Fertility effects of* Cardiospermum halicacabum *leaves have been evaluated in this study and the study showed that ALE of* C*.* halicacabum* has the ability to increase fertility of male Wistar rats.

It is well known that herbs have been used since the beginning of civilization to enhance and correct fertility. Administration of 100 mg/kg and 200 mg/kg body weight of aqueous extract of* C*.* halicacabum* for 30 days increased both caput and cauda epididymal sperm count in male rats. Sperm count is considered as an important parameter to assess the effect of chemicals on spermatogenesis [12]. Further, serum testosterone levels were increased in male rats treated with ALE. Hence, increase in sperm count can be attributed to the increase in plasma level of testosterone because this male hormone has significant contribution on initiation and maintenance of spermatogenesis [13]. These findings are comparable with the studies of Morakinyo et al. [14] with male rats treated with* Zingiber officinale* (ginger). It had been suggested that saponins present in plant extracts have the capability of increasing the body natural testosterone levels by raising the level of leutinizing hormones (LH). These LH released normally by the pituitary gland help to maintain testosterone levels; as LH increase, so does the testosterone [15]. Moreover, phytochemical analysis has shown that saponins are present in ALE of* C*.* halicacabum* [16]. Thus increased testosterone levels observed in the present study can be attributed to presence of saponins in the plant extract.

Motility is an important factor in the success of fertilization and any negative impact of motility would result in detrimental effects to fertilization ability [17]. Sperm acquires mobilization ability during their epididymal transit and the epididymis initiates the motility by providing unique microenvironment and also secreting proteins, which are important in initiating sperm motility [18]. In the present investigation the observed increased the motile spermatozoa and decreased immotile sperm in groups A and B. Similar results were observed with rats treated with bitter leaves [19] and* Telfairia occidentalis* leaves extract [20]. They suggested that increase in motility is due to increase of glucose metabolism resulting in production of pyruvate that is essential for the activity and survival of sperm cells. Further,* C. halicacabum* leaf has antioxidant property against Reactive Oxygen Species (ROS), which has toxic effect on sperm quality and function [21]. Available literature has confirmed that aqueous extract of* C. halicacabum* leaf has broad spectrum of bioactive compounds including tannin, flavonoid, terpenoids, and cardiac glycosides [8, 9]. Moreover, flavonoids present in this plant have been shown to possess many pharmacological properties including antioxidant activities and, hence, flavonoids also may have a contributory effect on its fertility properties and other pharmacological effects that the plant possesses [22].

Most changes of motility patterns were recorded in caput region and this might be due to an alteration in the microenvironment in the caput epididymis. However, it is important to mention that active compound of* C. halicacabum* (L.) has the ability to increase motility by accessing epididymes. Indeed, increased fertilizing ability observed in rats treated with* C. halicacabum* (L.) can be attributed to increased sperm count and sperm motility.

In a mating experiment, usually females are mated with treated males. In that experiment presence of implantation sites in females can be considered as evidence of successful implantation. If it happens, fertility test is considered to be positive [23]. The results presented in this paper also show that the ingestion* C. halicacabum* by adult male rats increased the number of impregnated females. The number of implantations and the number of viable fetuses were also increased. This effect may be due to increase in sperm motility and sperm density. Further, increase in testosterone levels may have led to increase in sexual competence of male rats.

The search for fertility enhancing agent with minimum side effects remains a challenge because higher plants with fertility effects are metabolically toxic. Treatment with high and low doses of* C. halicacabum* leaf extract did not exert any renotoxicity or hepatotoxicity indicating nontoxicity effect of the extract on general body metabolism. Interestingly, both high and low doses of* C. halicacabum* lowered the serum alkaline and acid phosphatases indicating hepatoprotective effect of the plant extract.

## 5. Conclusions

In conclusion, aqueous extraction of* Cardiospermum halicacabum* leaves increased fertility in male rats through increasing sperm motility, sperm count in a dose dependent manner, and this may be due to increased testosterone levels in the serum. Aqueous leaf extract also increased the number of females impregnated, number of implantations, and number of viable fetuses while decreasing the total number of resorption sites in the pregnant females. It also possesses a significant hepatoprotective effect indicating negative side effects. The increased fertility may be adduced to antioxidant activity due to flavonoids and increase in serum testosterone level, which may be due to saponin components.

## Figures and Tables

**Table 1:** Epididymal, testicular, and seminal vesicle weight after 30 days treatment.

	Epididymal weight (mg)	Testis weight (mg)	Seminal vesicle weight (mg)
Group A	0.1593 ± 0.005	2.4698 ± 0.014	0.5434 ± 0.034
Group B	0.1567 ± 0.006	2.4935 ± 0.016	0.5680 ± 0.034
Group C	0.1488 ± 0.005	2.4478 ± 0.015	0.6230 ± 0.032

Data are expressed as mean ± S.E.

**Table 2:** Total sperm count (×10^−6^) in caput and cauda epididymal regions of rats treated with 200 mg/kg (group A), 200 mg/kg ALE (group B), or distilled water (group C).

Groups	Total sperm count of caput epididymis (×10^−6^ mL)	Total sperm count of cauda epididymis (×10^−6^ mL)
Group A	245.25 ± 2.74^∗∗^	312.53 ± 12.37^∗∗^
Group B	228.47 ± 8.90^∗∗^	294.94 ± 5.03^∗∗^
Group C (control)	104.93 ± 7.54	224.54 ± 7.92

Data are expressed as mean ± S.E. ^∗∗^ *p* < 0.01.

**Table 3:** Sperm motility in caput and cauda regions after 30 days treatment with 200 mg/kg (group A), 100 mg/kg (group B) aqueous extraction of *Cardiospermum halicacabum* leaves, and the distilled water (group C).

	Immotile sperm (%)	Twitching sperm (%)	Motile sperm (%)
Caput region			
Group A	40.25 ± 2.74^∗^	14.62 ± 3.26^∗^	38.60 ± 5.03^∗∗^
Group B	44.47 ± 5.38^∗^	15.89 ± 2.05^∗^	28.99 ± 3.26^∗∗^
Group C (control)	46.93 ± 1.29	24.57 ± 0.60	18.44 ± 1.26

Cauda region			
Group A	52.94 ± 3.16	8.75 ± 0.98	188.15 ± 3.39^∗∗^
Group B	51.23 ± 4.30	10.12 ± 0.51	157.58 ± 4.41^∗∗^
Group C	51.30 ± 1.6	10.79 ± 0.81	106.79 ± 2.26

Data are expressed as mean ± S.E. ^∗^ *p* < 0.05, ^∗∗^ *p* < 0.01.

**Table 4:** Serum testosterone levels (mg/mL) of male rats treated with 200 mg/kg (group A), 200 mg/kg (group B) aqueous extract of *C. halicacabum*, or distilled water (group C) for 30 consecutive days.

Groups	Testosterone (mg/mL)
Group A	4.25 ± 0.74^∗∗^
Group B	3.88 ± 0.99^∗^
Group C (control)	2.93 ± 0.54

Data are expressed as mean ± S.E. ^∗^ *p* < 0.05, ^∗∗^ *p* < 0.01.

**Table 5:** Fertility study of Wistar rats repeatedly treated for 30 days with 200 mg/kg (group A), 100 mg/kg (group B) aqueous extraction of *Cardiospermum halicacabum* leaves, and the distilled water (group C).

Treatment	Number of pregnant females	Number of implantation sites	Number of viable fetuses	Total number of resorption sites
Group A	19/20	9.96 ± 1.83^∗^	9.85 ± 1.67^∗∗^	4^∗^
Group B	18/20	9.19 ± 1.05^∗^	8.73 ± 1.12^∗∗^	5
Group C (control)	17/10	7.17 ± 0.60	6.23 ± 1.05	6

Data are expressed as mean ± S.E. ^∗^ *p* < 0.05, ^∗∗^ *p* < 0.01.

**Table 6:** Serum levels of alkaline phosphates, acid phosphates, ALT, AST, GGT, and total proteins in animals treated with 200 mg/mL (group A), 100 mg/mL (group B) ALE, and the control (group C).

Serum parameters	Group A	Group B	Group C (control)
Reno toxicity			
Serum urea (g/dL)	15.25 ± 2.10	15.91 ± 1.50	17.36 ± 1.73
Creatinine (g/dL)	0.26 ± 0.21	0.31 ± 0.06	0.26 ± 0.09
Total protein (g/dL)	5.85 ± 0.99	6.98 ± 0.20	6.79 ± 0.51

Hepatotoxicity			
Alkaline phosphates (U/L)	159.24 ± 13.90^∗^	161.22 ± 18.54^∗^	189.63 ± 22.10
Acid phosphates (U/L)	71.31 ± 8.71^∗^	78.51 ± 11.67^∗^	93.60 ± 13.96
ALT (U/L)	21.60 ± 3.38	21.20 ± 2.20	23.53 ± 2.64
AST (U/L)	22.04 ± 4.40	24.69 ± 6.69	22.86 ± 8.13
GGT (U/L)	6.43 ± 1.37	7.63 ± 0.74	8.34 ± 1.54

Data expressed as mean ± SE. ^∗^ *p* < 0.05.

## Abbreviations

ALE: Aqueous leaf extract
ALT: Alanine aminotransferase
AST: Aspartate aminotransferase
GGT: Gamma-glutamyltransferase.
